# The Mobile Patient Information Assistant (PIA) App during the Inpatient Surgical Hospital Stay: Evaluation of Usability and Patient Approval

**DOI:** 10.3390/healthcare11050682

**Published:** 2023-02-25

**Authors:** Magdalena Görtz, Antonia Wendeborn, Michael Müller, Markus Hohenfellner

**Affiliations:** 1 Department of Urology, University Hospital Heidelberg, 69120 Heidelberg, Germany; 2 Junior Clinical Cooperation Unit ‘Multiparametric Methods for Early Detection of Prostate Cancer’, German Cancer Research Center (DKFZ), 69120 Heidelberg, Germany; 3 Medical Faculty, Ruprecht-Karls University of Heidelberg, 69117 Heidelberg, Germany; 4 mbits Imaging GmbH, 69115 Heidelberg, Germany

**Keywords:** electronic health, hospital, medical app, mobile health, surgery, telemedicine

## Abstract

Mobile eHealth apps are becoming increasingly important tools in healthcare management, capable of providing education and support at any time. There is little knowledge about surgical patients’ appreciation and use of these apps. The objective of this study was to develop and evaluate a user-friendly medical app (PIA; Patient Information Assistant) for providing individual patient information before and after inpatient urological surgery. Twenty-two patients aged 35 to 75 years were provided with timely information, push notifications, and personalized agendas (e.g., date of presentation, time of surgery, time of doctor’s consultation, imaging appointment) via the PIA app. Of the 22 patients, 19 evaluated the PIA app in terms of usage and usability, benefits, and potential for improvement. Of the study participants, 95% did not need any assistance to use the app, 74% confirmed that the PIA app made them feel better informed and more satisfied with their hospital stay, and 89% stated that they would like to re-use the PIA app and support the general use of medical apps in healthcare. Thus, we created an innovative digital health information tool, allowing targeted support for doctor–nurse–patient communication and offering great potential for patient support before and after surgery. Our study revealed that use of an app during the surgical hospital stay is readily accepted and benefits patients by acting as an additional informative tool.

## 1. Introduction

The healthcare industry is on the precipice of widespread digitization. The last decade brought great increases in electronic health record adoption and attestation to the use of defined documentation practices that are key to realizing the value of technology-enabled healthcare [1]. Health information technology describes the hardware, software, users, implementation, inputs, data, and outputs of computerized systems in the healthcare delivery environment. It takes many forms in healthcare organizations, such as patient health records, patient portals, computerized physician order entry systems, electronic prescribing, lab and radiology information systems, clinical data repositories, business intelligence applications, and health information exchanges [2]. The increasing adoption of various health information technologies has created new channels for doctor–patient communication, especially when the communication of information is critically important, such as during cancer therapy. By improving access to care, health information technology solutions were proven to enhance care delivery efficacy and make patients feel more confident and empowered, which supported their active involvement in the process, creating better relationships with the healthcare team [3]. Other promising areas of health information technology applications are the use of telehealth for pediatric patients with medical complexity [4] and the use of wearable healthcare devices for the self-monitoring of geriatric patients on a real-time basis [5]. Health information technology does offer the potential for transforming the healthcare delivery system and improving the health of patients, including those with urologic diseases [2].

In 2012, the European Commission announced the eHealth Action Plan 2012–2020 as an agenda to empower patients and healthcare workers, link up devices and technologies, and invest in research towards the personalized medicine of the future [6]. Because of its ease of use and broad acceptance, mHealth is considered to be a valuable tool in the implementation of patient-centered care, which has become a central goal of healthcare systems and international standards [7]. Precise definitions of eHealth and mHealth vary because they are applied in differing contexts and because of the ongoing advancements of information technologies that lead to new applications for eHealth and mHealth [8]. However, eHealth can be defined broadly as “the use of information and communications technology, especially the Internet, to improve or enable health and healthcare” and mHealth, which is a subdivision of eHealth, as “medical and public health practice supported by mobile devices, such as mobile phones, patient monitoring devices, personal digital assistants, and other wireless devices” [9]. mHealth includes all telecommunication devices for the transfer of healthcare information between participants at different locations, especially when referring to services that are mobile and wireless [10]. eHealth and mHealth tools have the potential to improve the quality of healthcare and reduce healthcare costs [11]. Educating patients with timely medical information delivered via their smartphones improves their levels of knowledge, treatment adherence, satisfaction, and clinical outcomes, as well as having positive effects on healthcare economics [12]. mHealth tools have further value because only a limited amount of new medical information can be correctly processed by the patient after a consultation, and mHealth apps can be used at any time and in any place [13,14]. This is particularly relevant in surgical treatment: healthcare professionals provide patients with information about treatment options, outcomes, how to prepare for surgeries, and advice for the recovery phase. Apps allow patients to revisit comprehensive medical information as often as they need, and push notifications allow healthcare providers to actively educate patients with timely information when details become relevant [12].

Consequently, ongoing advances in smartphone technology and healthcare apps could play important roles in enhancing patient-centered care in the future. There are currently over 300,000 mHealth apps available in the Apple App Store and Google Play Store [15]. An important motivator for health app users is the accessibility of specific information that can increase their knowledge about their conditions. However, it is important to ensure that this information is up-to-date, trustworthy, and valid [16]. Despite the increasing mHealth market, there is a lack of involvement of healthcare professionals in the design of medical apps, including in urological surgery [17]. Previous research showed that the number of trustworthy and high-quality apps on offer is extremely low. In former studies, most of the available medical apps were considered inappropriate for use in daily clinical practice, or their purpose had not been evaluated in studies [18]. In Germany, a previous multicenter study reported that, so far, only a minority of the population used medical apps. In detail, even as much as approximately 90% of the patients owned a smartphone, while only 11% of the patients reported previous medical app usage, and only 3.5% of the patients received an app recommendation from a physician [19].

With the PIA (Patient Information Assistant) app, we developed a user-friendly mobile app for patients in need of inpatient urological surgery to provide high-quality, timely information in the perioperative period. The purpose of the app was to be a 24-hour companion to help prepare patients for hospitalization, enhance patient knowledge about the ward routine, and encourage patients to follow postoperative behavioral recommendations at home. The main goals of this study were to assess the user-friendliness and the patient’s acceptance of the PIA app, as well as positive and negative user experiences.

## 2. Materials and Methods

### 2.1. App Development

The PIA app was developed in cooperation between the Urology Clinic of Heidelberg University Hospital and the software developer mbits imaging GmbH. The design and implementation process of the PIA app was based on well-established structured software design principles. First, the basic needs and requirements from the perspective of the clinicians were assessed by contextual inquiries with the different stakeholders. Contextual inquiries are a tool within the software design process in which a potential user of the system is interviewed on the distinctive steps of the corresponding real-world process [20]. The notable idea of the contextual inquiries is to decouple the interviewee from the technical aspects of the software that will be developed. By employing this tactic, requirements are recorded solely on the causal and temporal conditions of a real-world process rather than on technical implementations that are often confused with user needs.

Second, a specification was created from the process definition which described the different parts of the app. The specification, therefore, contained a formal description of the tech stack that would be used alongside with implementation details such as the program flow and the user interface of the app. To address the importance of an adequate user interface for patients, several sketches were created and reworked prior to any implementation.

All stakeholders and the development team refined the specification draft during several main meetings until there was a common agreement on a final version. Doctors from the Urology Clinic at Heidelberg University Hospital were the initiators and drivers of the app development, and they played a major role in determining the content of the app. All content of the app was designed with the experience of the urological doctors and nurses at the respective hospital who knew what patient information was important, often forgotten, or frequently requested. The content of the PIA app was created specifically for the various urological surgeries conducted in the hospital by the medical team.

The pure implementation took approximately two weeks of full-time work by a team of two experienced developers. The whole process was accompanied by several meetings with clinicians to review different implementation versions. Briefly, there were three main alpha versions of the app or “release candidates” until a final version was reached. The versions were tested on a small, selected cohort of patients before the study was conducted. The feedback and improvement recommendations from these test runs were incorporated into the development of the final version of the PIA app. The final version was then validated by the clinicians, i.e., whether it could fulfil the real-world process requirements. Afterwards, the deployment of the PIA app in the app stores was initiated. The development was completely financed by the company mbits imaging GmbH to explore and evaluate the business case of an app that supports patients’ surgical journey.

The time of the whole development process—which started with a kick-off meeting and ended with the release of the first app version in the respective app stores—was approximately six months.

### 2.2. Software Technology

The PIA app was built using two major software technologies: “Flutter” [21] and “Firebase” [22]. Flutter provided software tools for creating apps on all operating systems, especially mobile platforms, while ensuring compliance with modern user interface guidelines. Firebase was a Google technology that represented a “backend as a service” platform. The platform, therefore, provided functionality for authentication, data validation, push notifications, and scalable storage of data of any kind in one system. The location of the physical storage of data could be chosen within the platform. For the PIA app, all data were stored on German servers.

Furthermore, the communication between the PIA app and the Firebase database occurred in real time and was encrypted at all times, which ensured live updates of messages while protecting them from unauthorized access.

To comply with General Data Protection Regulation requirements, the PIA app provided all functions required for the patient to make use of their proper rights: a privacy statement describing the information collected and how it was processed, and the processed data could be viewed, transferred, and deleted at any time.

### 2.3. Recruitment of Participants and Questionnaire

Fifty consecutive patients with planned admissions for inpatient urological surgery at the Urology Clinic of Heidelberg University Hospital were screened in April 2021. The inclusion criteria of the study were that the patient was admitted for surgery on a scheduled basis, used a smartphone in their daily life, was an adult capable of giving consent, and had sufficient German language skills. The app was provided to the study participants from their first presentation at Heidelberg University Hospital on the day of surgical preparation until several weeks after the inpatient surgery. The study participants were asked to download the PIA app for free onto their smartphones from Google Play Store for Android and from Apple App Store for iOS.

As there is no established, standardized questionnaire for the evaluation of medical apps, despite the continuous development of new apps [6], a questionnaire was developed for the evaluation of the PIA app. In designing the questionnaire, the MAUQ questionnaire for patient apps was used as a guideline [6] and adapted for the PIA app. This questionnaire included the categories of general patient information, information about the hospital stay, satisfaction with the app, evaluation of the user interface as well as applicability. Patients were asked to fill out a first questionnaire containing demographic characteristics upon study inclusion. A second questionnaire was provided to be filled out at the end of the app’s use after discharge, including closed-ended questions about usability, usage data, and perceived benefits, and open-ended questions about the strengths and weaknesses of the PIA app. Important values were satisfaction, supportiveness, reusability, and recommendability of the app.

The PIA app was provided as an alternative source of information in the study. The standard education and information provided by physicians and nurses of Heidelberg University Hospital was maintained for the participants in the study. Push messages about the latest updates or developments in the ward routine were sent to the patients by an undergraduate (AF) under direct doctors’ supervision. Timely information was obtained by involving key hospital staff in the conduct of the study (including ward physicians, surgeons, surgical coordinators, nursing management). The entire study implementation and the sending of personalized push notifications to the patients were continuously supervised by MG.

### 2.4. Ethical and Legal Framework

Data were collected prospectively, and the study was approved by the Ethical Committee of the University of Heidelberg (Approval No. S-778/2019). All study participants gave their informed consent. Patient participation was voluntary; the study did not change the standard therapy, but merely supplemented it. Consent could be withdrawn by the patient at any time, without giving reasons and without disadvantages for further medical care. In the event of withdrawal from the study, data obtained would be deleted at the request of the patient. The names of patients and all other confidential information were subject to medical confidentiality and the provisions of the General Data Protection Regulations. After registering and accepting the privacy policy in the PIA app, patients could log into the app with their email address and user password. The PIA app used a multilevel security concept in which, ultimately, only the user could access the data on their smartphone using a password of their choice. In addition, end-to-end asymmetric data encryption was used, data were pseudonymized after collection, and (encrypted) data were automatically deleted after completion of the study.

### 2.5. PIA App Content

Hospitals and operating rooms are logistically complex to run. The more insight patients are given into the hospital’s procedures and regulations, the better they can adapt to the processes. When background information regarding medical interventions is given to patients, they better understand the reasonableness of the recommendations, and treatment adherence is increased. The medical team, on the other hand, can be relieved from the need to repetitively explain the same standard information to every patient (e.g., the procedures on the ward) through an app, which would give them more time for the individual engagement with the patient. In addition, for patients, a hospital stay is often an exceptional situation, which leads to them forgetting information that was communicated verbally. As a result, operations may have to be cancelled (e.g., if patients have accidentally continued to take their anticoagulants) or the maximum success of the operation cannot be achieved (e.g., because patients forget post-operative behavioral instructions).

**Figure 1:**
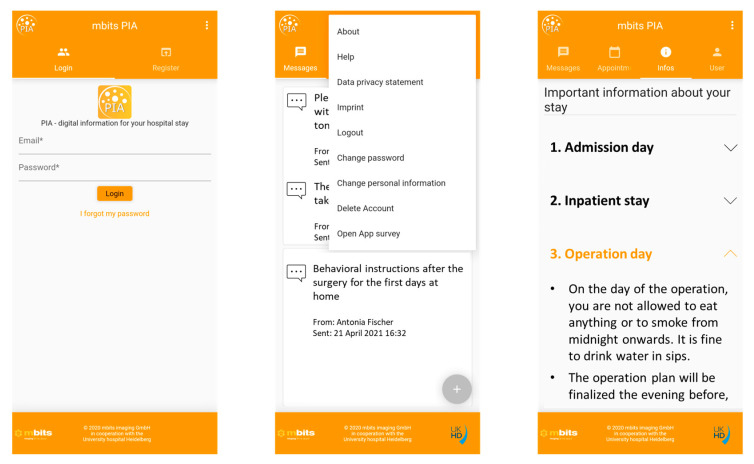
Structure, design, and functionality of the PIA app.

The PIA app was designed to provide the necessary information during the hospital stay as well as preoperative information to help prepare for hospitalization, and discharge information for the rehabilitation process at home. The app was intended to be a 24-hour companion to provide optimal patient information and to improve patient knowledge and self-management. (Figure 1). The PIA app features had the aim of an optimized patient education and information flow, with a smaller knowledge deficit for the patients before and after medical treatment. Information tailored to the patient was to be made accessible at any time and in any place.

The PIA app contained three tabs: one for general information, one for a personal agenda, and one for push notifications. The content of the app was offered in German.

General information in the app included, among others, an overview of the required pre-operative examinations (e.g., anesthesia, electrocardiography), items to carry to the hospital, a location plan in the hospital, a daily schedule for the ward (doctor’s visits, doctor’s time for discussions with relatives, visiting hours), latest COVID-19 rules, an introduction to the medical team, the use of multimedia in the patient’s room, the preparation for the surgery including storage of valuables, post-operative medication including pain killers, contact details of support services (inside and outside the hospital), and information about the social services.

Regarding the personal agenda, patients received perioperative appointments in a calendar in the PIA app, such as date and place of presentation in the hospital, time of surgery, date and place of imaging appointments, time of doctor’s consultation for discharge examinations, date and place of re-presentation in the hospital.

A few examples of push notifications that were sent as a reminder or update to the patients included: which and when long-term medication was necessary to discontinue, at what time point it was needed to fast before surgery, timely information about delays in the surgical schedule, what the goals were for the first post-operative days, why specific examinations were carried out, what to remember at home after the respective operation, when to present again in the hospital for follow-up, what the long-term behavioral recommendations were to avoid a recurrence (e.g., stone metaphylaxis, pelvic floor training).

Sample screenshots of the mobile health application PIA demonstrate the user experience and highlight the app’s functions. After the patient logged in, the PIA app offered three tabs: one for general information, one for individual push notifications, and one for a personal agenda. The app pages were translated into English; the original app tabs can be found in the Supplementary Material.

## 3. Results

### 3.1. Study Population

**Figure 2:**
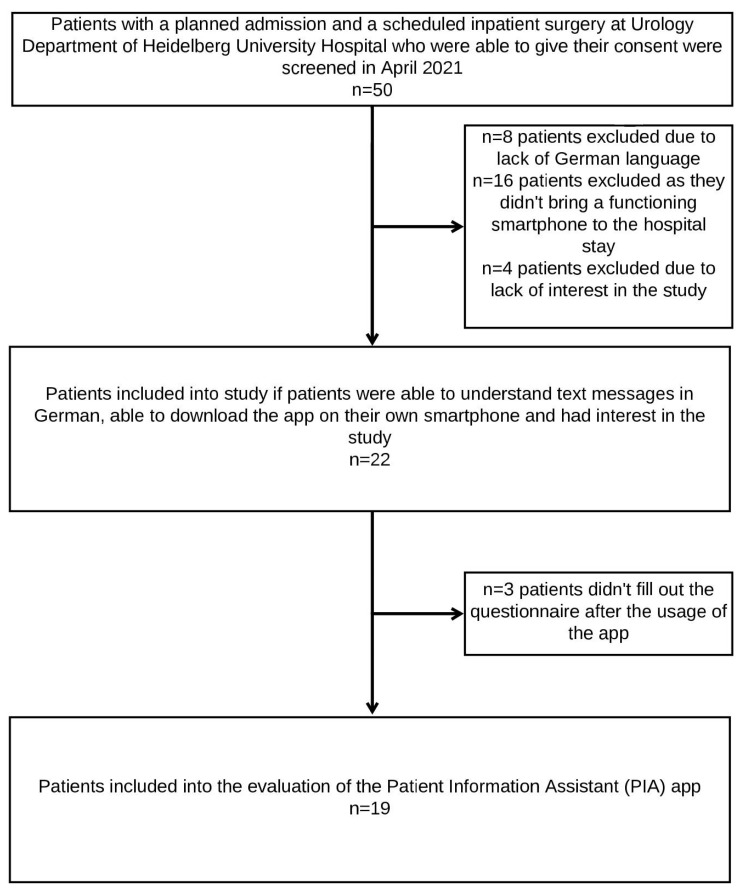
Flow chart for inclusion of study participants.

Common reasons for exclusion of patients from the proof-of-concept study were not carrying a smartphone to the hospital, lack of German language, or lack of interest in the study (Figure 2). Nineteen of the twenty-two patients who were provided with the newly developed PIA app evaluated the app at the end of the usage period. They evaluated the PIA app via a questionnaire assessing functionality, suitability, and utility from a patient perspective. The median age of the study cohort was 50 years old; the youngest study participant was 35 and the oldest was 75 years old. In total, five out of nineteen patients were female and fourteen out of nineteen patients were male. The median length of hospital stay was 5 days (Table 1). The most common surgeries were tumor removal from the kidney or prostate, and kidney stone removal.

### 3.2. Patient Evaluation of the PIA App

**Figure 3:**
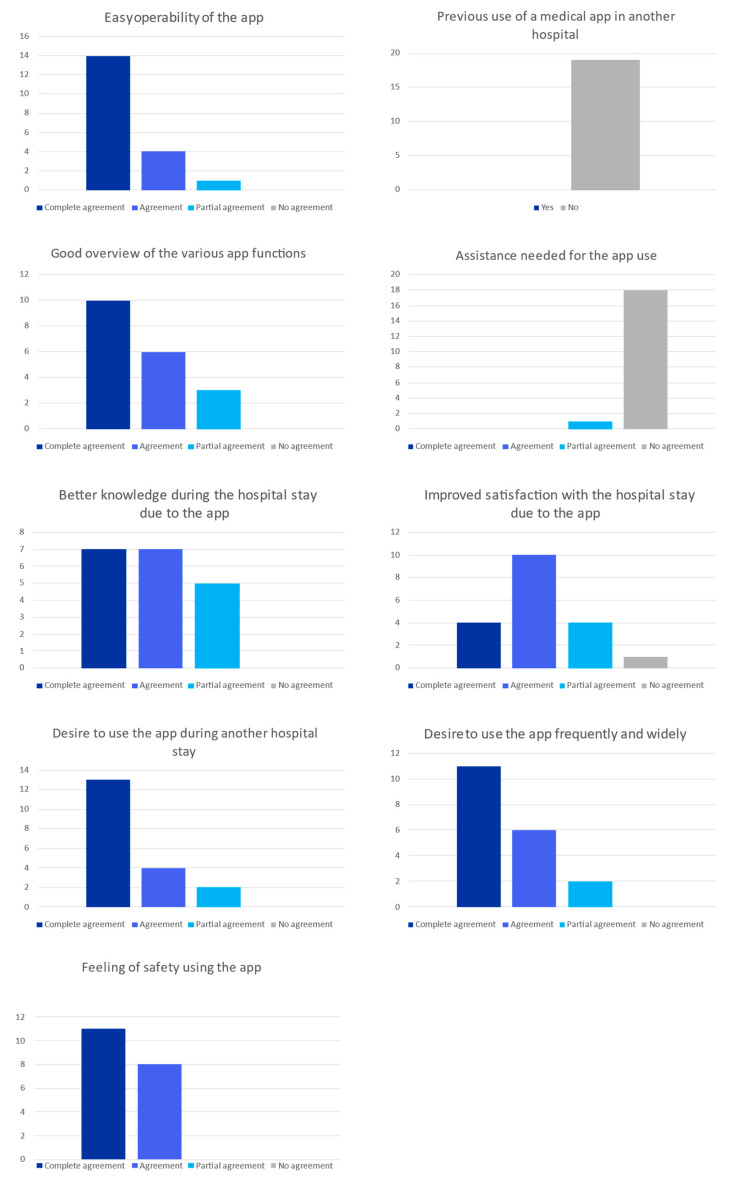
Evaluation of patient satisfaction with the PIA app.

Regarding usability, the study participants confirmed the easy operability of the PIA app, with 18 out of 19 patients (95%) confirming that the app was easy to use (fourteen patients completely agreed, four patients agreed, one patient partially agreed, and none disagreed). Similarly, 18 out of 19 patients (95%) did not need any assistance to begin using the PIA app (Figure 3). The structuring of the various functions of the app was found to be comprehensible and clear (ten patients completely agreed, six patients agreed, three partially agreed, and none disagreed; topic “good overview of the various app functions” in Figure 3). The aim of this question was to ask the patients about the interface of the app and the organization of the information in the app, so that they could easily find the information they needed.

In terms of innovation, none of the patients had previously used a medical app in another hospital. None of the patients had any safety concerns about using the PIA app, and all the study participants felt secure during the study period, in the way that they believed that the data they shared in the app was securely stored (eleven completely agreed, eight agreed, and none partially agreed or disagreed; topic “feeling of safety using the app” in Figure 3).

In terms of benefits for the patients, fourteen out of nineteen patients (74%) agreed or completely agreed that the provision of the PIA app made them feel better informed during their hospital stay, with five out of nineteen patients (26 %) partially agreeing and none disagreeing. Fourteen out of nineteen patients (74%) stated that they were more satisfied with their overall hospital stay because of the provision of the PIA app. The study participants expressed that they would like to use the PIA app in a further hospital stay (thirteen patients completely agreed, four patients agreed, two patients partly agreed, and none disagreed). In general, the study patients said that they would like to use the PIA app regularly in healthcare (eleven patients completely agreed, six patients agreed, two patients partly agreed, and none disagreed).

The evaluation of the app was obtained after use via structured questionnaires. A total of 19 out of 22 recruited patients gave feedback about the PIA app, which is shown above. The participants’ answers are given as the number of patients who chose each multiple-choice answer.

**Figure 4:**
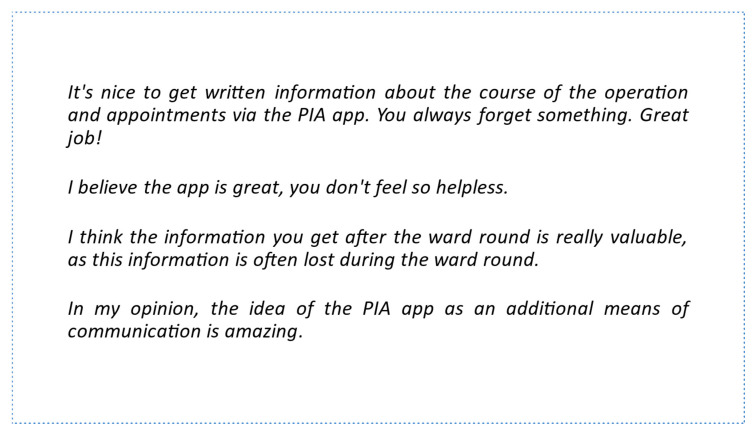
Individual patient feedback on the PIA app.

A total of 13 of the 19 study patients who filled out the questionnaire completed the free-text evaluation. In the free-text evaluation, suggestions for improvements from the study’s participants included delivery of push messages to the mobile phone in standby mode, directly receiving the results of examinations via the app (e.g., blood tests), and receiving more detailed descriptions on planned interventions. Further feedback about the PIA app is illustrated in Figure 4.

## 4. Discussion

Patients reported a general lack of readily available information, e.g., on medical procedures, side effects, and the logistics of care [23,24]. mHealth holds great potential in healthcare as a low-threshold offer to inform patients in the hospital, and as a companion at home before and after treatment.

In this proof-of-concept project, we developed and evaluated, to the best of our knowledge for the first time, a user-friendly app to provide patients with timely information before and after inpatient urological surgery. Our study revealed several major results.

First, the use of the PIA app was both highly innovative and desirable for patients. Of the fifty patients screened in this study, only four were not included due to a lack of interest. Another 24 patients could not be included in the study because they did not speak sufficient German or did not carry a functioning mobile device with them. None of the study participants used a medical app in another hospital before; however, after using the PIA app, they clearly expressed their recommendation for the increased use of the PIA app in healthcare. Second, our study results confirmed that the use of the PIA app was unproblematic and did not require a need for assistance. Third, the evaluation results revealed that patients had no privacy or security concerns about the digital technology of the medical app PIA. There are not just potential benefits by mHealth applications, but patients can also have severe direct and indirect privacy concerns and the fear of discrimination; for example, through the employer or health insurance company. Potential difficulties for the acceptance of mHealth applications are the patients’ concerns about data security, the consequences of potential data theft, and data sales to third parties. Users of medical apps rely on the ethical operation of app services and need to trust the apps they use [25]. Fourth, the vast majority of patients confirmed that the provision of the PIA app made them feel better informed and that they were more satisfied with their overall hospital stay because of the provision of the PIA app.

More and more patients, as well as hospital staff, possess a smartphone. By adding the concept of timing to patient education, it is possible to increase the likelihood that patients will receive the right information at the right time [12]. The digitalization of the healthcare sector can help to create healthcare that is more predictive, preventive, personalized, and participatory [26], while simultaneously relieving medical staff of the need to repetitively provide standard information to the patients, ensuring that they can spend their time on more individual engagements with patients. The more efficient use of information and personnel can contribute to long-term cost reductions as well as the optimization of work processes at hospitals. Smartphones and their apps are appealing to healthcare providers, as they provide intuitive user interfaces and allow for easier interaction with patients [27], particularly in the postoperative outpatient setting. mHealth can facilitate virtual means of contact, which is a viable solution when surgical patients live in remote areas, and in situations such as pandemics (e.g., COVID-19) [15]. Thus, innovative apps, such as the PIA app for urological surgery patients, have the potential to create benefits for patients, physicians, hospitals, and public health.

Within the scope of mHealth, smartphones are increasingly being utilized for patient education, disease prevention, medical diagnosis, and disease monitoring. The COVID-19 outbreak has further led to the acceptance of new strategies such as telemedicine as pragmatic approaches and alternatives to doctor consultations [28,29]. Successful examples of mHealth interventions include medication adherence (e.g., improving the cardiometabolic profile of patients with hypertension, or monitoring the opioid use of patients with chronic noncancer pain) and promoting smoking cessation [30,31,32]. Further examples of mHealth applications include follow-up assessments after hospital discharge and inpatient stroke rehabilitation [33] and timely postoperative care education after total knee replacement [34]. In urological settings, the feasibility of an app for postoperative behavioral recommendations after radical prostatectomy was demonstrated previously [35].

The PIA app distinguished itself from previous works by its timely support of surgical patients not only in the outpatient setting but also during the hospital stay. In other medical disciplines, there are already promising research results on patient support provided by medical apps during the inpatient hospital stay. For example, to address the challenges of communication and remote rounding during COVID-19, a mobile app was developed to communicate changes in hospital policies and enable direct telephonic communication between clinical team members and hospitalized patients in terms of inpatient telehealth [36]. In another study, to facilitate the daily life of the physician and patients with diabetes, medical apps to improve the inpatient glycemic management were described. The applications identified for in-hospital glycemic management promoted better glycemic mean and lower risk of hypoglycemia than usual management [37]. In a psychiatric inpatient unit, a recent study suggested that there is significant untapped potential for utilizing smartphone applications for psychiatric monitoring and treatment, with a majority of patients expressing interest in using mental health apps in the future [38]. These research results demonstrate that the use of health applications to support inpatient therapy is on the rise to be incorporated into medical practice with user activity metrics suggesting that mHealth solutions will remain important. In line with our study results, medical apps can be tools to improve the quality of patient care and can have sustained value among clinicians in communicating with inpatients [36,37,38]. However, the number of studies is still scarce.

Going further, envisioning several dimensions for expanding the PIA app in subsequent projects is straightforward. Now that we have confirmed the usability and patient approval of the PIA app in our initial proof-of-concept cohort, the next step is to investigate the benefit of the app in a prospective randomized study. The potential added value and increased productivity should be evaluated by patients, doctors, and nursing staff alike. Connecting the PIA app to existing hospital information systems through the Internet of Things has the potential to further optimize timely patient information and can be a step towards a smart hospital [39]. In the near future, we expect eHealth and mHealth to become revolutionary in healthcare. Major industry directions are health data curation and enrichment, AI-enabled diagnostic interpretation, and an application marketplace for healthcare providers [40]. Real-time monitoring devices can gather live patient data from sensors/wearables and send inputs to a mobile medical app on a smartphone, server, or network to support clinical decision making [17]. This new wave of big-data-powered personalized risk assessment tools can help healthcare professionals to make better, more individualized decisions. The standardized capture of patient-reported outcome data with minimal resource utilization over an mHealth application can be used for quality assessment, optimization, and research. mHealth will likely assist in the transition from isolated healthcare sources with inaccessible proprietary information to an integrated paradigm of continuous care built around the individual, with an improvement in health outcomes and a reduction in costs [15].

The limitations of our study include that this study was conducted solely in the field of urology surgery in the German healthcare system. It was limited to 22 German-speaking patients in a single inpatient surgical hospital in Germany. Our analysis of the PIA app was an initial exploratory study focusing on the evaluation of the app’s usability and quality. As there has only been little research on mHealth applications for inpatient support, the exact requirements and level of acceptance of a medical app for inpatient surgical patients were unknown. The present proof-of-concept study allowed us to assess the integrability of the PIA app into daily clinical routine, to evaluate its usability and approval, to become aware of problems that could occur during implementation, as well as to contribute to the evidence base. A successful initial proof-of-concept clears the way for setting up larger clinical studies and to establish the PIA app in clinical routine [41]. So far, the widespread use of mobile apps on smartphones and tablets has not become reality in inpatient hospital care in German hospitals. Initial evidence of potential benefits from mHealth apps is required to overcome challenges that hinder the wider adoption of apps (e.g., regulatory, financial, and organizational issues). First promising descriptive results enable a process to implement, supervise, and evaluate clinical mHealth apps in larger cohorts [42].

Because of the limited scope of application in our study, the gained evidence was only representative for the respective target group. For instance, population groups such as non-German speakers or patients with visual issues did not meet study requirements and were not included. Thus, of the 50 screened patients, only 22 took part in the study, as the other patients did not carry a mobile device to the hospital, did not speak sufficient German, or were not interested in the study participation, resulting in selection bias. With the small size of this proof-of-concept study, the generalizability of our findings to the overall population must be handled with particular caution. This necessitates the need to explore any additional requirements that may be needed for medical apps in emerging nations or more rural nations and healthcare systems, particularly as it relates to technology literacy and internet access. A future mixed-methods project could be conducted to gain a broader perspective on user experience and usability of the PIA app by involving participants from various cities, states, and countries. A mixed-methods research design would systematically combine quantitative and qualitative methods for a comprehensive understanding of the research questions. It would enable to move beyond ‘what works’, documented by measurable parameters in quantitative research, and understand ‘what works for whom, why, how, and when’, through qualitative research in different populations [43]. Moreover, it is crucial to evaluate whether the requirements of patients for mHealth apps vary in disciplines other than surgery.

A further limitation of our study includes the utilized survey tool and the challenge of applying validated criteria for the evaluation of medical apps. The assessment of the PIA app focused on obtaining patient feedback through a survey; however, studies that rely on self-reporting of patients are particularly prone to recall bias. In addition, the questionnaire, which was applied in the study, and which was developed based on the MAUQ questionnaire for patient apps [44], has not yet been validated. Future directions may include the design of validated instruments to evaluate the vast diversity of mHealth formats.

Finally, it must be emphasized that some patients may not own a smartphone and cannot be reached via mHealth apps. This is changing with smartphone ownership on the rise; however, no patient should be left out. It is important to be aware that mHealth tools are designed to support and enhance, but they will probably never fully replace direct consultation with a healthcare professional.

## 5. Conclusions

Via the introduction of the medical app PIA, we created an innovative digital health information tool that offered great potential for patient assistance before and after surgery and for targeted support of doctor–nurse–patient communication in the hospital. The German patients of our exploratory proof-of-concept study approved the use of the PIA app during their surgical hospital stay, as the provision of the app made them feel better educated about hospital procedures and medical background. The broad majority of the study participants confirmed the easy usability of the app and supported the general future use of the PIA app in clinical routine.

## Figures and Tables

**Table 1:** Study population.

Study Cohort	
Patients evaluating the app, n	19
Female participants, n	5
Male participants, n	14
Age, yr ^1^, median (IQR ^2^)	50 (44–57)
Length of hospital stay, days, median (IQR)	5 (4–7)
Endourological intervention	6
Robot-assisted, minimally invasive surgery	6
Open surgical intervention	7

^1^ yr = year; ^2^ IQR = interquartile range.

## Data Availability

Not applicable.

## Supplementary Materials

The following supporting information can be downloaded at: https://www.mdpi.com/article/10.3390/healthcare11050682/s1. Structure and design of the PIA app in German.
