# Supplementary material for: The Mobile Patient Information Assistant (PIA) App during the Inpatient Surgical Hospital Stay: Evaluation of Usability and Patient Approval
Source: Healthcare (Basel). 2023 Feb 25;11(5):682. doi: 10.3390/healthcare11050682 (PMC10000762; doi:10.3390/healthcare11050682)

Supplementary Material – Structure and design of the PIA app in German.

The structure of the PIA app for German-speaking patients is shown.

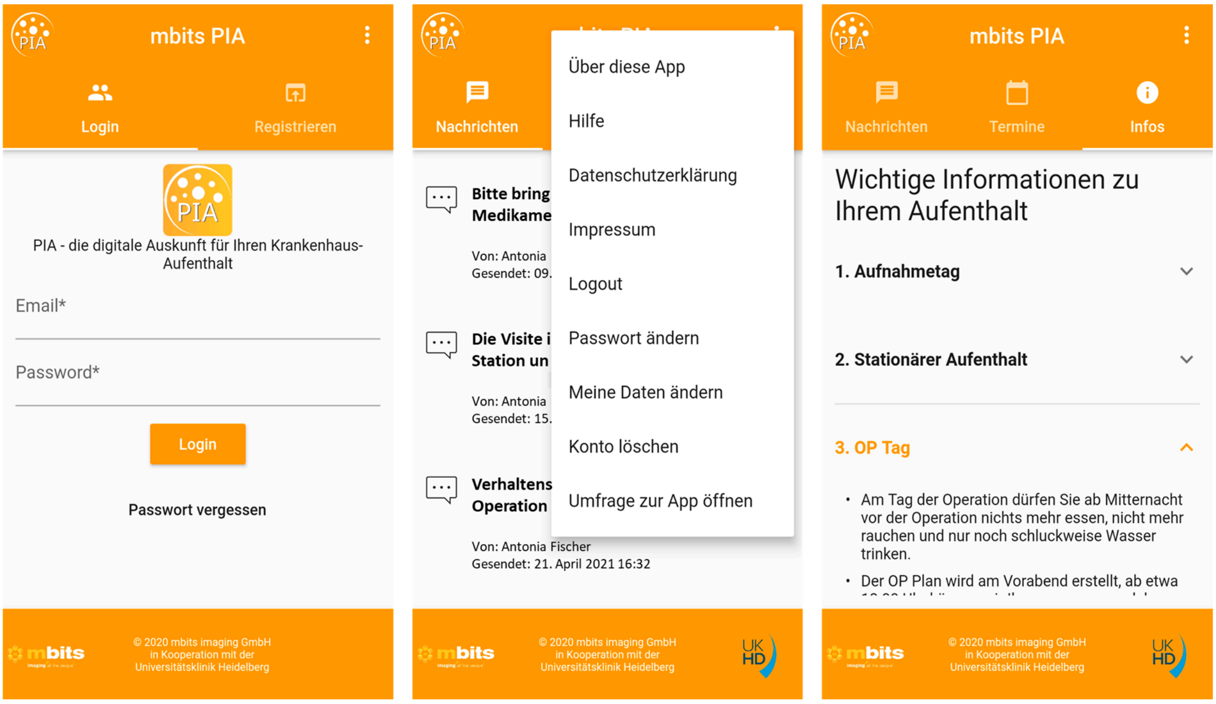

Supplement: Supplementary file 1 [file healthcare-11-00682-s001.zip › healthcare-2154191-supplementary.pdf]
